# Research Progress of Microtransfer Printing Technology for Flexible Electronic Integrated Manufacturing

**DOI:** 10.3390/mi12111358

**Published:** 2021-11-03

**Authors:** Li Zhang, Chong Zhang, Zheng Tan, Jingrong Tang, Chi Yao, Bo Hao

**Affiliations:** Key Laboratory of Vibration and Control of Aeronautical Power Equipment of Ministry of Education, Northeastern University at Qinhuangdao, Qinhuangdao 066004, China; zhangchongsci@163.com (C.Z.); t15308012725@163.com (Z.T.); 2001jacktheripper@gmail.com (J.T.); Y201910293345@163.com (C.Y.); zlcyj09a20@163.com (B.H.)

**Keywords:** flexible electronics, microtransfer printing, mechanical model, PDMS seal, SMP seal, ink

## Abstract

In recent years, with the rapid development of the flexible electronics industry, there is an urgent need for a large-area, multilayer, and high-production integrated manufacturing technology for scalable and flexible electronic products. To solve this technical demand, researchers have proposed and developed microtransfer printing technology, which picks up and prints inks in various material forms from the donor substrate to the target substrate, successfully realizing the integrated manufacturing of flexible electronic products. This review retrospects the representative research progress of microtransfer printing technology for the production of flexible electronic products and emphasizes the summary of seal materials, the basic principles of various transfer technology and fracture mechanics models, and the influence of different factors on the transfer effect. In the end, the unique functions, technical features, and related printing examples of each technology are concluded and compared, and the prospects of further research work on microtransfer printing technology is finally presented.

## 1. Introduction

**Figure 1:**
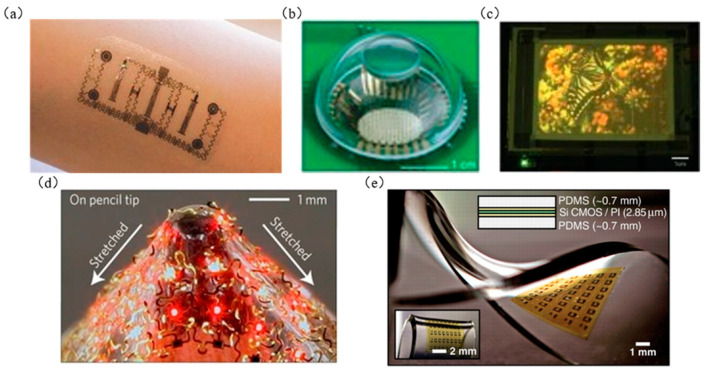
Examples of flexible electronics: (**a**) electronic skin (reproduced from [49]), (**b**) flexible bionic electronic eye, (reproduced from [50]), (**c**) flexible display (reproduced from [51]), (**d**) optical image of AllnGaP μ-lLEDs (6 × 6) array transferred to PDMS substrate and stretched on the pencil (reproduced from [52]), and (**e**) stretchable and foldable Si-CMOS circuit (reproduced from [53]).

As an emerging micro-/nanomanufacturing technology for flexible electronics, microtransfer printing has many advantages [1,2,3]. For example, microtransfer printing has an extensive range of target substrates, including flexible and stretchable polymers, glass, ceramics, metals, and metal foils [4]. Numerous “inks” can be composed of almost any material, including metal [5,6,7,8,9], inorganic material [10,11,12,13,14,15], organic material [16,17], carbon [18,19], and colloid [20,21], as well as a variety of ink forms, from molecules to macroscopic materials and fully integrated equipment [20,22]. It can transfer semiconductor materials and is a controllable, repeatable, and efficient printing process [23,24,25,26]. It is expected to realize high precision and large-scale rapid integration of heterogeneous material arrays [19,27,28,29]. By increasing the number or shape of microstructures on the seal, the printing process can be achieved efficiently [30]. The microtransfer printing technology with the above characteristics provides a desirable manufacturing method for wearable electronic products [4,31], electronic skin [32,33,34,35,36], scalable electronic products [37,38], micro-LED monitor products [16,30,39], flexible sensors [4,40,41,42,43], and flexible monitors [4,44,45,46,47]. Figure 1 shows some flexible electronic products manufactured by using microtransfer printing technology.

**Figure 2:**
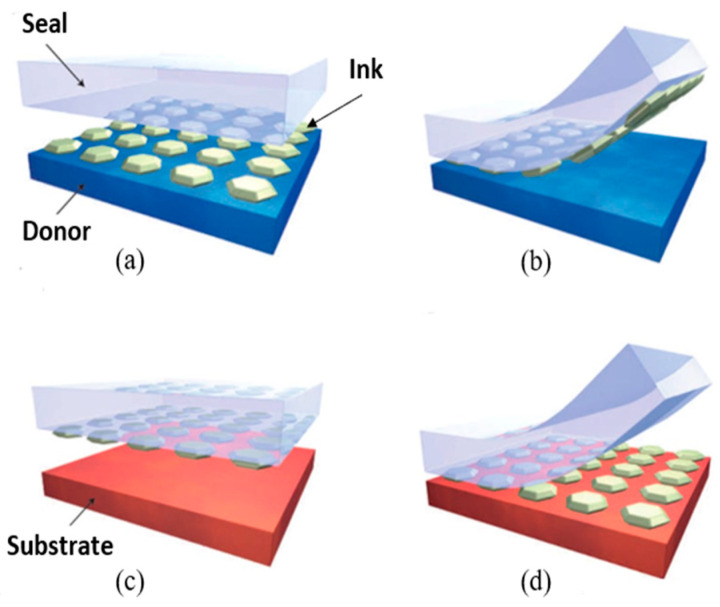
Microtransfer printing assembly process diagram (reproduced from [48]).

Rogers et al. first proposed this kind of heterogeneous integration method with deterministic and high-speed operation [48]. It can be called microtransfer printing technology. The basic process of this technology mainly includes extracting ink from the donor substrate and printing ink on the target substrate. Figure 2 illustrates the basic process of transfer printing. During the process of transfer printing, the ink on the target substrate is adsorbed by using an elastic seal. This step requires that the edges of both the seal and the target substrate are aligned and in contact. Transfer the seal with ink onto the target substrate, align, and contact the seal with the target substrate, after which the ink will be printed onto the target substrate. In the above process, to ensure the mutual edge alignment between the seal and the donor substrate and the target substrate is to ensure the accuracy of the transfer printing effect and improve the transfer printing efficiency. A strong seal/ink adhesion force is required in the process of ink adhesion of the seal to ensure that the seal extracts ink from the donor substrate. A weak seal/ink adhesion force is required in the process of ink release of the seal to ensure that the ink is releasing to the seal. Therefore, the pivotal factor of the transfer printing process is adjusting the surface adhesion force of the seal.

The seals used in microtransfer printing technology are mostly polydimethylsiloxane (PDMS) polymers (Sylgard 184, Dow Corning) [48]. PDMS has the characteristics of flexibility [30], conformal contact with solid substrates, optical transparency [54], and low mechanical properties. It is an important feature of the PDMS seal widely used in microtransfer printing technology. With the development of microtransfer printing technology, new seals, such as shape memory (SMP), TRT tape, and water-soluble tape (PVA), have been produced. The chemical and physical properties of the seal, such as material composition [55], elastic modulus [56], glass transition temperature [4,57], and surface microstructure [58,59,60,61,62,63], are closely related to the adhesion required for transfer printing. Table 1 summarizes the characteristics of each type of seal applied by transfer printing technology involved in this review and calculates the corresponding adhesion force. Finally, it lists the adhesion control mechanism of each transfer printing technology.

For the transfer seal (PDMS), the most important property is the elastic modulus, which is mainly reflected in the ability of the seal to resist elastic deformation under external force [30]. Choi et al. [64] adjusted the elastic modulus of PDMS by changing the mixing ratio of the curing agent and utilized the method to study the influence of microtransfer printing of the elastic modulus of the seal, which ultimately yielded smaller elasticities. Guo et al. [30] studied the relationship between the width and the elastic modulus. In addition, they concluded that the elastic modulus of PDMS is also related to the mixing ratio of the curing agent, curing temperature, curing time, and accuracy of the measuring instrument. During the transfer printing process, if the elastic modulus of the seal is lower, the seal generated is severely deformed by an external force, which will eventually lead to transfer printing failure. Therefore, it is of significance to explore the elastic modulus of the seal for microtransfer printing [65]. Ferreira et al. [4] used laser heating to induce the thermal mismatch at the seal/ink interface to produce free expansion and, finally, to facilitate the release of ink from the seal [59]. However, this method requires a high temperature (e.g., 250–300 °C), but the high temperature will damage the temperature-sensitive seals and substrates. In addition, high-power lasers can create problems, such as experimental safety and expensive equipment. Feng et al. [57] developed a new type of SMP seal with a glass transition temperature of T_g_ ≈ 43 °C, which solves the problem of damage to the seal and targets the substrate through high-power laser. Furthermore, the researchers designed a seal with microstructures by observing biological structures, such as octopus suckers [58], the tentacles of an aphid [61], and the soles of a gecko [66,67], and used the seal to achieve adhesion switching. With the in-depth research on microtransfer printing seals, a variety of transfer printing methods have gradually emerged, such as laser-controlled seal temperature transfer printing and microstructured seal-assisted transfer printing.

Lu et al. [20] came up with a new type of surface-treatment-assisted switchable transfer printing method, which can easily manufacture the specified pattern on a PDMS film. Song et al. [39] developed a laser-guided programmable noncontact transfer printing technology, which controls the temperature of an elastic seal containing a film-sealed cavity structure inside through laser energy to achieve contactless transfer printing. As a new type of seal, shape memory polymer (SMP) materials have the characteristics of shape fixation and shape recovery, which make the structural design of the seal more open [68]. Multiscale stereoscopic inks with arbitrary shapes can be clamped and manipulated singly, deterministically, or in a large number of selective ways [69]. The optimal transfer printing technology should have easily controllable interface adhesion, while SMP seals are usually shaped by changes caused by the parameter of temperature, which makes it easier to control the adhesion of SMP seals [68]. Song et al. attempted to use SMP materials for transfer printing technology, which broke through the challenge of multiscale ink transfer printing for arbitrary shapes. Kim et al. [68] used SMP for deterministic microassembly to provide dynamic stiffness control by controlling the temperature of the shape memory polymer (SMP), which can greatly provide adhesion in the extraction in ink engineering.

The purpose of this review is to introduce the latest advances in microtransfer printing technology, a new way to manufacture flexible electronic devices, and focus on the working principles of seven microtransfer printing technologies. The layout of this review is as follows: First, it introduces the classifications of the existing microtransfer printing technology and makes a detailed analysis of each method. These categories mainly include kinetic control transfer printing and laser control seal temperature transfer printing. Then it summarizes this review and looks forward to the future of micro-transfer printing technology.

## 2. Classification of Microtransfer Printing Methods

The critical factor that determines the efficiency and success rate of microtransfer printing technology is the achievement of controlled interfacial layering at the seal-to-ink interface and the ink-to-target substrate interface. Therefore, the controllable seal adhesion force is a hot topic in microtransfer printing technology. Most researchers control the adhesion of elastic seals through the separation speed of the seal interface, the loading method, the microstructure of the sealing surface, and the temperature of the seal, which gives birth to the following microtransfer printing methods: kinetic control transfer printing [37,48,70,71,78,79,80], laser control seal temperature transfer printing [4,39,57,59,68,74,81], microstructure seal to assist in transfer printing [60,61,62,82], transfer printing with applied shear load [73,83,84], tape-assisted transfer printing [76,77], transfer printing with a seal inflatable [85], and magnetic control transfer printing [56,72].

### 2.1. Kinetic Control Transfer Printing

**Figure 3:**
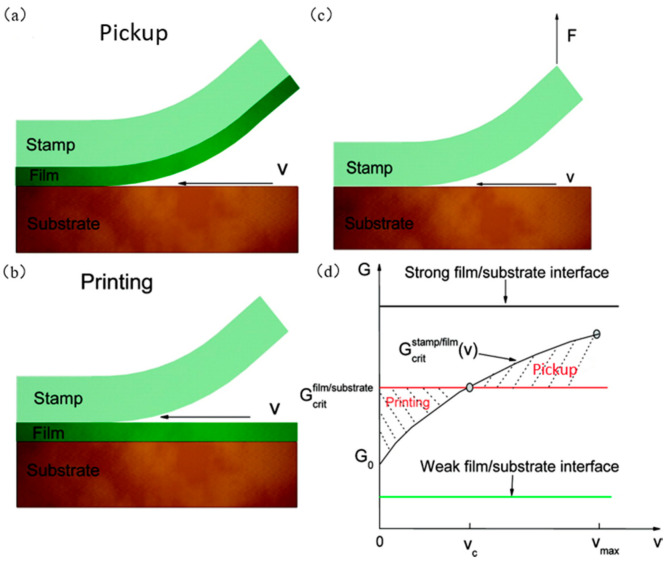
Kinetic control transfer printing process diagram. (**a**) Mechanical model diagram of the film extraction process (reproduced from [69]), (**b**) mechanical model diagram of the printing film process (reproduced from [69]), (**c**) schematic diagram of the separation of the seal under vertical upward pull F interface (reproduced from [69]), and (**d**) relationship between energy release rate and peeling speed (reproduced from [69]).

**Figure 4:**
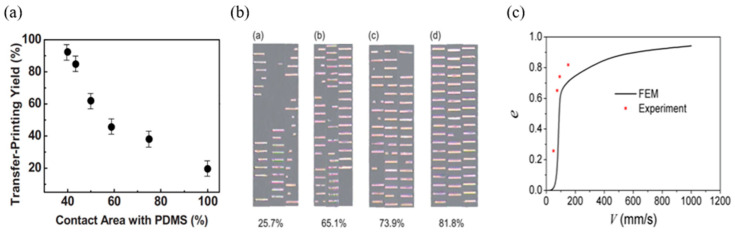
Kinetic control transfer printing principle. (**a**) The relationship between the percentage of the seal contact area and the percentage of the successful transfer printing area (reproduced from [71]), (**b**) optical image of transferred Si tape under different peeling speeds of the PDMS seal (reproduced from [37]), and (**c**) graph of transfer printing efficiency and peeling speed (reproduced from [37]).

Rogers et al. [48] were the first to propose a kinetically controlled transfer printing method. This method uses velocity to dynamically control the adhesion force switching between interfaces to achieve transfer printing, because of the adhesion characteristics of PDMS. The energy release rate $G_{\mathrm{crit}}^{\mathrm{stamp/film}}$ of the seal/ink interface depends on the layering speed ν, while the energy release rate $G_{\mathrm{crit}}^{\mathrm{stamp/film}}$ of the ink/target substrate interface is independent of velocity. The main process of kinetic control transfer is as follows: The ink is extracted from the donor substrate by using the adhesion characteristics of the seal through a relatively large stripping speed (about 10 cm/s, i.e., $G_{\mathrm{crit}}^{\mathrm{film/substrate}}<G_{\mathrm{crit}}^{\mathrm{stamp/film}}$ [78]). The seal and ink are contacted with the target substrate, and the seal is extracted at a relatively small stripping speed (about 1 mm/s, i.e., $G_{\mathrm{crit}}^{\mathrm{film/substrate}}>G_{\mathrm{crit}}^{\mathrm{stamp/film}}$ [78]). Additionally, the ink is transferred to the target substrate. Figure 3a,b shows the mechanical model diagrams of the extracting film and printing film processes. Feng et al. [78] regarded the seal/substrate interface separation as a steady-state crack propagation process. Figure 3c shows the schematic diagram of the seal interface separation when subjected to a vertical upward pull F. The energy release rate is

(1) $$G=\frac{F}{w}$$

where *G* is the energy release rate, *F* is the tensile force, and w is the width of the seal. Formula (2) is the energy release rate of the seal/film interface, which is

(2) $$G_{\mathrm{crit}}^{\mathrm{stamp/film}}\left(v\right)\;=G_{0}\left[1+{\left(\frac{v}{v_{0}}\right)}^{n}\right]$$

where $G_{0}$ is the critical energy release rate, ν is the stripping speed, $\nu_{0}$ is the reference stripping speed, and n is the scaling parameter determined during the experiment. Formula (2) applies to various stripping speed ranges, temperature ranges, metal/polymer interfaces, polymer/polymer interfaces, and so forth. Figure 3d shows a graph of the relationship between energy release rate and stripping speed, where $\nu_{max}$ is the maximum stripping speed and ν is the critical stripping speed. The critical speed depends on the stiffness of the seal. Under the condition of determining the geometric dimension of the seal, the critical speed decreases with the decrease in the modulus of the seal [78]. Feng et al. [78] also studied the effect of temperature on the transfer printing structure, which found that low temperatures favored the extraction of the ink and high temperatures favored the transfer printing. Also explored is the discontinuous film, both discrete of distribution ink. The relation between average energy release rate and contact area is

(3) $$G_{\mathrm{pr}\mathrm{int}}=fG^{\mathrm{stamp}/\mathrm{film}}(v)$$

where ƒ represents the contact area fraction (0 < ƒ < 1) of the seal/ink interface, where $G^{ink/stamp}\left(\nu \right)$ is the energy release rate. Formula (3) shows that the adhesion strength between the seal/ink/target substrate interfaces is proportional to the contact area. Kim et al. [71] conducted a 90° stripping experiment on this basis and concluded that reducing the contact area was beneficial to improve the success rate of transfer printing. Figure 4a shows the relationship between the percentage of the seal contact area and the percentage of the successful transfer area. Jiang et al. [37] also conducted an experimental investigation and simulations on seal transfer printing Si tapes with different stripping speeds. It was found that the transfer printing efficiency increases with the increase in the stripping speed, but the transfer printing efficiency increases slowly when the stripping speed is greater than 100 mms^−1^. Figure 4b shows the optical image of the transferred Si tape under different stripping speeds of the PDMS seal, and Figure 4c shows a graph of transfer printing efficiency versus stripping speed in the simulation experiment. The simulation results are consistent with the experimental results.

**Figure 5:**
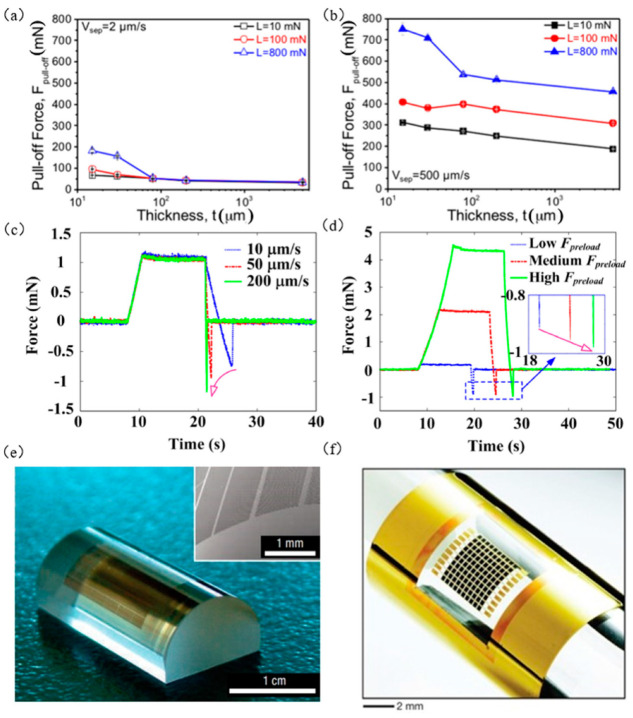
Kinetic transfer printing principles and examples. (**a**,**b**) The relationship between the thickness of the seal and the tensile force under different contact loads at different peeling speeds (reproduced from [80]), (**c**) the relationship between the pulling force and the peeling speed (reproduced from [70]), (**d**) the relationship between tension and preload (reproduced from [70]), (**e**) printed array formed on cylindrical glass lens (reproduced from [48]), and (**f**) flexible gallium arsenide solar cell array (reproduced from [86]).

Since the controllable range of bonding is narrow, in most situations, kinetic control transfer printing requires an adhesive to be applied above the target substrate to assist in the transfer printing process. However, this method cannot change the adhesive force of the seal and make the transfer printing process continuous, which greatly reduces the efficiency of the transfer printing. Kim et al. [80] found that by changing the design parameters of the seal and adjusting the stripping speed, the controllable range of adhesion strength can be effectively extended, and also high yield manufacturing can be achieved. Figure 5a,b shows graphs of the relationship between seal thickness and tensile force under different contact loads, L=10 mN, L=100 mN,L=800 mN, at the stripping speeds $V_{sep}=2\;\mu m/s$ and $V_{sep}=500\;\mu m/s$, respectively. The results show that the stripping force is correlated with the critical interface contact area, and the thicknesses of the seal, contact load, and stripping speed are the main influencing factors. Liang et al. [70] established a tensile force model in which the stripping speed and pretightening force were considered. Finally, the relationship between stripping speed, pretightening force, and tensile force was studied. From Figure 5c, we can know that the tensile force increases with the increase in peeling speed at a certain preload. From Figure 5d, we can know that the tensile force increases with the increase in pretightening force at a certain stripping speed. It follows that a larger preload force should be applied during the transfer extraction phase, and a smaller preload force should be applied during the printing phase, which can play a significant role in improving the transfer printing output. Through analysis of the theoretical model and experimental data, the results show that the pretightening force at a large stripping speed has a greater influence on the tensile force than the pretightening force at a small stripping speed [70]. Figure 5e,f shows examples of transfer printing on cylindrical glass lenses and PET substrates using the kinetic control transfer printing method.

The process of kinetic control transfer printing is simple and easy to operate, but the stripping speed control adhesion strength range is limited, the transfer printing is greatly restricted, and its durability depends on the preparation of the target substrate and the cleanness and flatness of the substrate surface. This method is also not applicable to target substrates of viscous materials. Therefore, its application is limited by different materials. Precise speed control is required during operation, which requires a speed control device to improve transfer printing efficiency, but greatly increases the cost of the transfer printing. Moreover, this transfer printing method has not produced an example of nanotransfer printing because the energy release rate is influenced by the contact area.

### 2.2. Laser Control Seal Temperature Transfer Printing

**Figure 6:**
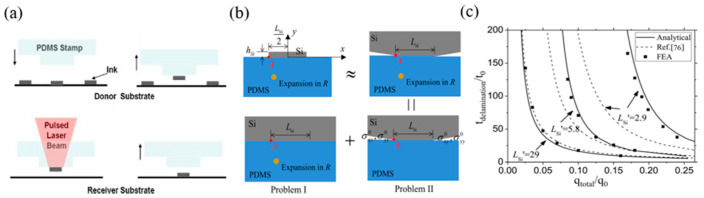
Laser-controlled seal temperature transfer printing technology. (**a**) Laser-controlled seal temperature transfer printing diagram (reproduced from [4]), (**b**) accurate interface fracture mechanics model diagram (reproduced from [59]), and (**c**) the peeling time scale rule of laser-controlled seal temperature transfer printing (reproduced from [59]).

Saeidpourazar et al. [4] developed a laser-controlled seal temperature transfer printing technique by exploiting the differences in the thermodynamic parameters (coefficient of thermal expansion and thermal conductivity) of PDMS seals and inks. The specific process of this technology is shown in Figure 6a. The seal with a microstructure selectively picks up the ink on the donor substrate and then transfers the printing of the ink above the target substrate. Due to the transparency of the PDMS seal to the laser (805 nm), the pulsed laser glow focuses on the ink through the seal. The ink transmits heat to the PDMS seal through the seal/ink interface, raising the temperature of the ink and the seal. Due to the thermodynamic differences between the seal and the ink, finally, the seal/ink interface is layered to realize transfer printing. Li et al. [81,87] established the thermodynamic theoretical model shown in Figure 6a, which did not consider the influence of silicon wafer size. The relationship for the delamination time $t_{\mathrm{delamination}}$ seal/silicon interface is calculated as follows:

(4) $$\frac{t_{\mathrm{delamination}}}{t_{0}}=f\left(\frac{q_{\mathrm{total}}}{q_{0}}\frac{L_{\mathrm{silicon}}}{L_{0}}\right)$$

In Formula (1),

(5) $$t_{0}=\frac{c_{\mathrm{silicon}}^{2}\rho_{\mathrm{silicon}}^{2}h_{\mathrm{silicon}}^{2}}{c_{\mathrm{PDMS}}\rho_{\mathrm{PDMS}}\lambda_{\mathrm{PDMS}}}$$

(6) $$q_{0}=\frac{\lambda_{\mathrm{PDMS}}}{\alpha_{\mathrm{PDMS}}}\sqrt{\frac{\gamma }{\mu_{\mathrm{PDMS}}}{\left(\frac{c_{\mathrm{PDMS}}\rho_{\mathrm{PDMS}}}{c_{\mathrm{silicon}}\rho_{\mathrm{silicon}}h_{\mathrm{silicon}}}\right)}^{3}}$$

(7) $$L_{0}=\frac{c_{\mathrm{silicon}}\rho_{\mathrm{silicon}}h_{\mathrm{silicon}}}{c_{\mathrm{PDMS}}\rho_{\mathrm{PDMS}}}$$

where $q_{\mathrm{total}}$ is the total heat of the seal/silicon interface irradiated by the pulsed laser, $h_{\mathrm{silicon}}$ and $L_{\mathrm{silicon}}$ are the thickness and width of the silicon wafer, $c_{\mathrm{PDMS}}$ and $c_{\mathrm{silicon}}$ are the specific heat, $\rho_{\mathrm{PDMS}}$ and $\rho_{\mathrm{silicon}}$ are the mass density, $\lambda_{\mathrm{PDMS}}$ is the thermal conductivity, $\alpha_{\mathrm{PDMS}}$ is the coefficient of thermal expansion, and $\mu_{\mathrm{PDMS}}$ is the shear modulus and the adhesion strength of the interface γ. The thermodynamic model does not apply to smaller silicon wafer sizes. Gao et al. [59] considered the influence of silicon wafer size and established an accurate interfacial fracture mechanics model (as shown in Figure 6b), which was used to derive the composite stress intensity factor, interfacial energy release rate, and critical delamination time, which eventually coincided with the finite element calculations, as shown in Figure 6c.

**Figure 7:**
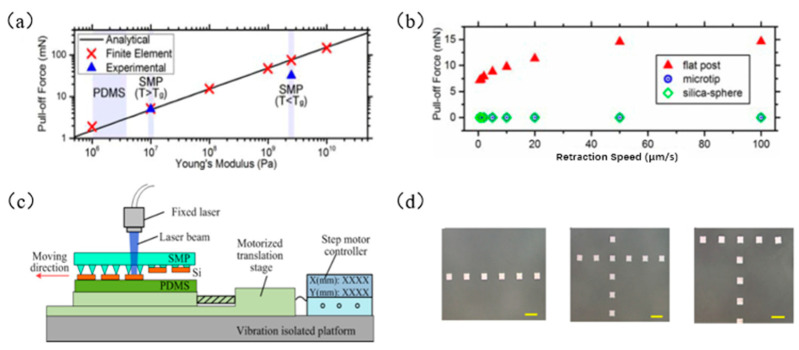
The principle of laser-controlled seal temperature transfer printing technology. (**a**) The relationship between the elastic modulus and the plane stress of the seal (reproduced from [68]), (**b**) the relationship between the two types of seals and the plane stress at different peeling speeds (reproduced from [68]), (**c**) laser programmable selective transfer printing equipment setup diagram (reproduced from [57]), and (**d**) examples of successful transfer printing of straight, cross, and T-shaped patterns (reproduced from [57]).

Eisenhaure et al. [68] took advantage of the SMP materials, which are rigid when the polymer is below the transition temperature T_g_ and can increase the adhesion strength of the seal. By replacing the traditional PDMS seal with an SMP seal, there are more options for seal design, and the analytical formula of seal plane stress is obtained:

(8) $$F_{pull-off}=\sqrt{25.31\gamma_{0}\left(2E_{SMP}\right)L^{3}}$$

where *E* is the modulus of elasticity, *L* is the width of the seal, and $\gamma_{0}$ is the adhesive strength. The finite element analysis is consistent with Formula (8), as shown in Figure 7a. They also compared the two seal surfaces (smooth seal surface and pyramid microstructure seal surface), which are more suitable for transfer printing. Figure 7b shows the relationship between the two types of seals and the plane stress at different stripping speeds. It is shown that the seal with a microstructure on the surface is more suitable for transfer printing.

**Figure 8:**
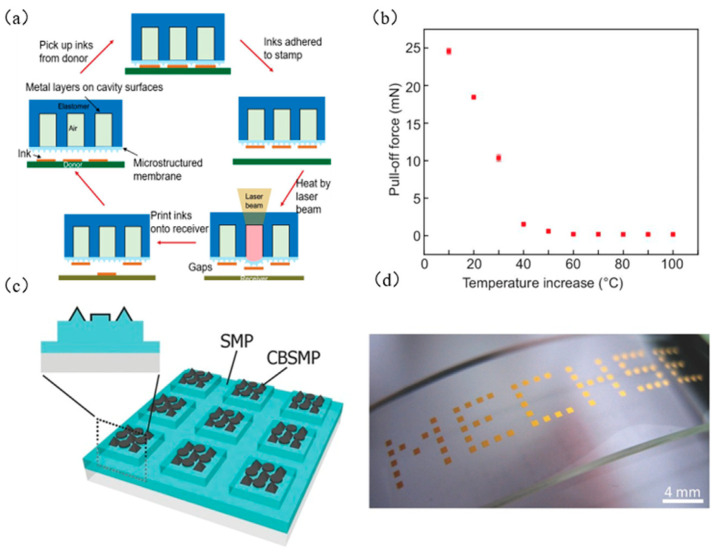
Laser-controlled seal temperature transfer printing technology process diagram and example. (**a**) Specific diagram of laser temperature control transfer printing technology for seal with cavity (reproduced from [39]), (**b**) stress relationship diagram at different elevated temperatures (reproduced from [39]), (**c**) SMP seal design drawing with carbon black composite material (reproduced from [74]), and (**d**) an example of selective printing of gold-plated silicon wafers that do not absorb infrared laser ink (MECHSE: Department of Mechanical Science and Engineering) (reproduced from [74]).

Feng et al. [57] developed a laser programmable selective transfer printing method that uses a stepper-motor-controlled precision platform to control the displacement of the target substrate and uses laser heating of the SMP seal to achieve a high conversion of the adhesion force. Figure 7c shows the setting diagram of laser programmable selective transfer printing equipment. Figure 7d shows examples of successful transfer printing of linear, cross, and T-shaped patterns. Song et al. [38] used SMP materials to design a seal with a circular cavity, with a metal layer on the upper surface of the cavity as a heat absorption layer, and closed the air inside the cavity with a film with a microstructure. The transfer printing process is shown in Figure 8a. Figure 8b shows the stress relationship diagram at different elevated temperatures. Kim et al. [74] prepared an SMP seal with a carbon black composite material (as shown in Figure 8c), which greatly improved the heat transfer rate by taking advantage of the material’s absorption of infrared laser light. At the same time, it also solves the limitation that the ink must be a laser-absorbing material for the laser transfer printing process and saves the complicated steps of calculating the laser power required for different ink materials and geometric shapes. Figure 8d shows an example of selective printing of gold-plated silicon wafers that do not absorb infrared laser ink.

Laser-controlled seal temperature transfer printing realizes noncontact transfer, eliminates the influence of the target substrate during the transfer printing process, and can transfer ink to any microstructured surface. The laser beam is used to heat the seal locally and instantaneously. Therefore, the laser-controlled seal temperature transfer printing has high selectivity, no hysteresis, and scalability and can achieve extremely high transfer printing efficiency. However, due to the need to reach a temperature sufficient to release the ink, the high temperatures will cause burns on the seal/ink interface and the target substrate. Therefore, it is necessary to accurately control the power of the laser pulse, and a higher-power laser can also generate safety problems. These problems will eventually result in expensive experimental equipment.

### 2.3. Microstructure Seal to Assist in Transfer Printing

Transfer printing technology usually requires tape, high pressure, or high-temperature methods to change the adhesion of the seal, but these treatment methods may harm the ink, seal, or seal interface. Inspired by the microstructures of gecko and insect tentacles, scholars created microstructure-seal-assisted transfer printing. The commonly used microstructures mainly include oblique columns, pyramids, and so on.

#### 2.3.1. Oblique Column Structure

**Figure 9:**
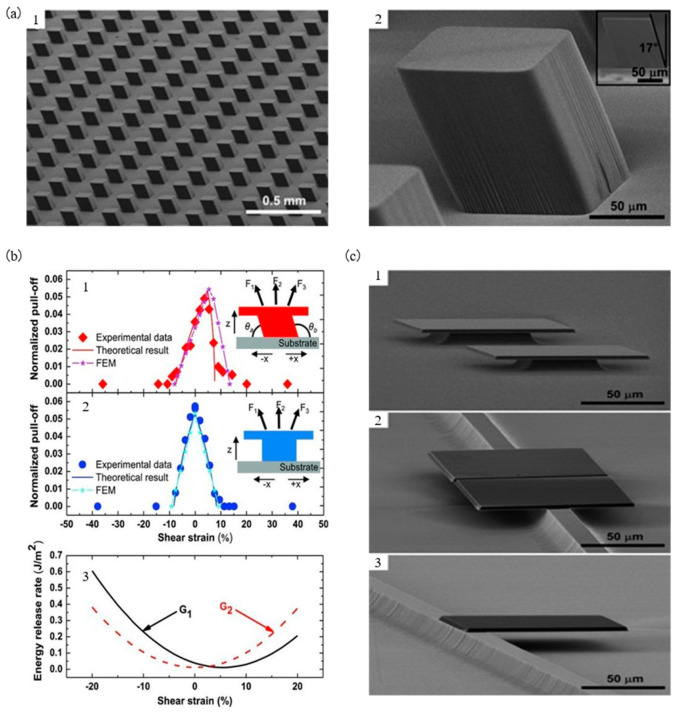
Assist transfer printing technology of a microstructured seal with an oblique column. (**a**) Scanning electron microscope (SEM) image of a seal made by PDMS (reproduced from [62]), (**a1**) is the microstructure array diagram of the seal with oblique columns, and (**a2**) is the characteristic diagram of the microstructure design of a single oblique column, (**b**) schematic diagram of the relationship between the adhesion strength of the seal and the shrinkage direction (reproduced from [62]), (**b1**) is the schematic diagram of the oblique pillar microstructure seal, (**b2**) is the principle diagram of the pillar microstructure seal, (**b3**) is the relationship graph between the energy release rate and the shear strain of the oblique pillar (G_1_) and pillar (G_2_) microstructure seals, and (**c**) successful example of seal transfer printing with an oblique column structure (reproduced from [62]), (**c1**–**c3**) respectively indicate that the micro device is transferred to the target substrate of the pillar, beam, and cantilever beam structure.

Rogers et al. [62] arranged the column microstructure with an angle (inclination angle of 17°) on the elastomer seal. The design of this structure realizes the height adjustment of the adhesion strength between the interfaces and provides transfer efficiency by allowing continuous rolling printing when using a cylindrical seal. Figure 9(a1) shows the scanning electron microscope (SEM) image of the column array on the surface of the seal, and Figure 9(a2) shows the specific design features of a single column structure. The angle of 17° inclination is selected mainly because the manufacturing process is relatively simple and has better adhesion performance. Figure 9b shows a schematic diagram of the relationship between the adhesion strength of the oblique column and the shrinkage direction. When the seal with the oblique column and the target substrate are in parallel contact with each other, two contact angles ($\theta_{a}$, $\theta_{b}$ and $\theta_{a}+\theta_{b}=180{}^{\circ}$) are generated. When a tensile force is applied to the vertical direction of the seal, due to $\theta_{a}<\theta_{b}$, it will break first from $\theta_{a}$ at the seal/target substrate interface. When the component force is applied in the opposite direction to the angle to pull the seal, the crack fracture at $\theta_{a}$ will be accelerated, resulting in a decrease in the tensile force ($F_{2}<F_{3}$). On the contrary, when the component force is applied to the same direction as the angle to pull the seal, the crack fracture at $\theta_{a}$ will be slowed down, resulting in increased tension ($F_{2}<F_{1}$). Therefore, in transfer printing, the extraction and transfer printing process is ready by selecting the conditions along and away from the angle. Figure 9(b2) shows the vertical seal in contrast to Figure 9(b1). Figure 9(b1,b2) shows the relationship between the normalized tensile force and the shear strain, where the standardized tensile force $P=F\left(EL^{2}\right)$, E, and L are the elastic modulus and width of the PDMS seal, respectively. By comparing the two figures, it can be concluded that the adhesion ability of the seal with an oblique column microstructure depends on the displacement direction.

Rogers et al. [62] also analyzed the mechanical model of interfacial delamination for seals with oblique column microstructures. The seal of height h is subject to vertical tension F and shear displacement μ applied to the bottom of the seal through the seal/target substrate interface. Tensile force F produces bending moment $Fh\cot\theta_{a}$ and shear strain $\gamma =\mu /\left(h+0.42L\right)$ on the seal. The bending moment generated by the shear force is $\mu \gamma hL^{2}$, in which the shear modulus of the seal is μ=E/3, E is the elastic modulus, and the total bending moment on the seal is

(9) $$M=Fh\cot\theta_{a}+\mu \gamma hL^{2}$$

The EL standardized crack tip energy release rate is given equivalently as F and the shear strain γ

(10) $$\frac{G_{1,2}}{EL}=0.113{\left(\frac{F}{EL^{2}}\right)}^{2}+0.0522\frac{h^{2}}{L^{2}}{\left(3\frac{F}{EL^{2}}\cot\theta_{a}-\gamma \right)}^{2}\pm 0.0928\frac{h}{L}\frac{F}{EL^{2}}\left(3\frac{F}{EL^{2}}\cot\theta_{a}-\gamma \right)$$

where subscripts 1 and 2, respectively, represent the crack at the seal $\theta_{a}$ and $\theta_{b}$ in Figure 9(b1). As shown in Figure 9(b3), when $\gamma \leq 3\frac{F}{EL^{2}}\cot\theta_{a}$, the energy release rate of the left crack is greater than that of the right crack. When $\gamma >3\frac{F}{EL^{2}}\cot\theta_{a}$, the energy release rate of the left crack is smaller than that of the right crack. When the energy release rate in Formula (10) is equal to the interface fracture toughness $\Gamma_{0}$, the crack starts to fracture. The critical tension is derived from Formula (10)

(11) $$\frac{F}{EL^{2}}=f\left(\frac{h}{L}\gamma ,\frac{h}{L}\cot\theta_{a},\frac{\Gamma_{0}}{EL}\right)$$

For the interface toughness $\Gamma_{0}=0.057\;N/m$, Formula (11) is consistent with the experimental data, as shown in Figure 9(b1). Figure 9c shows an example of successful transfer printing.

#### 2.3.2. Pyramid Structure

**Figure 10:**
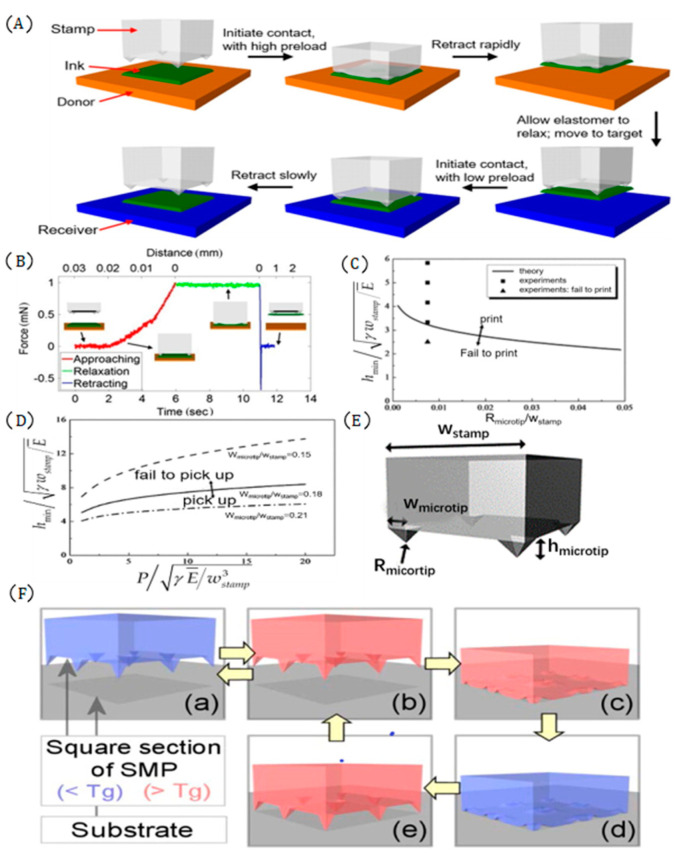
Assist transfer printing technology of the seal with a pyramid microstructure. (**A**) Transfer printing diagram with a pyramid structure seal (PDMS) (reproduced from [61]), (**B**) time graph of force in complete mechanical collapse at a retraction speed of 1 mm/s under a preload of 1 mN (reproduced from [61]), (**C**) the relationship between the height of the pyramid and the contact radius at the top of the pyramid (reproduced from [60]), (**D**) pyramid height and preload relationship diagram (reproduced from [60]), (**E**) seal size parameter diagram (reproduced from [60]), and (**F**) transfer diagram with a pyramid structure seal (SMP) (reproduced from [82]), The blue seal indicates that the seal is in a rigid state, and the red seal indicates that the seal is in a flexible state.

Huang et al. [61], inspired by aphids, designed an elastomeric seal surface with pressure-induced microstructure (pyramid shape) changes, which creates depressions and recovers its original shape when subjected to external pressure changes. It is the change of the seal surface that causes the corresponding change of adhesive strength, which provides another new method for the microtransfer printing technology. Huang et al. [61] explained the transfer printing process using a surface seal with a pyramid structure. The transfer printing process is as follows: During extraction, the downward directional preload makes the microstructure mechanically concave, which increases the contact area between the seal and the ink, thus increasing the adhesion strength between the interfaces. Because of the low adhesion strength between the ink and the donor substrate, the ink can be picked up at a higher speed. The ink is transferred to the target substrate, and when the elastic force on the surface of the seal is recovering, the adhesion strength between the ink and the seal interface gradually decreases with the contact area between the interfaces so that the ink and the target substrate completely contact. The specific process is shown in Figure 10A. Figure 10B shows the force-time diagram of complete mechanical collapse at a shrinkage speed of 1 mm/s under a preload of 1 mN. Two curves with a slope can be seen in the curve segment (red) of this graph. In the first segment of the curve, the slope characterizes the effective elastic constant of the microtip compression, where the deformation of the microstructure is not the main reason. The slope in the second curve represents the elasticity of the microstructure and its elastic support. The curve (green) segment indicates that it terminates for 5 s under a specified load of 1 mN, and then retracts at a speed of 1 mm/s. The curve (blue) segment indicates the retraction process. The sharp negative characteristic in the curve corresponds to the rapid release from the contact surface, and its magnitude defines the adhesion force.

The most important parameter in the transfer printing process of the surface seal using a pyramid structure is the height of the microstructure. This is because too much height will make the tip of the microstructure too sharp, resulting in the inability to pick up the ink. The adhesion of the very low microstructure is too strong, resulting in the inability to release the ink to the target substrate [60]. Wu et al. [60] constructed 2D and 3D mechanical models of the pyramid structures and explored the height range of the microstructure for successful transfer printing. Based on the 3D mechanical model, the minimum and maximum heights of the obtained microstructure are:

(12) $$h_{\min}\approx \sqrt{\left[3.04\ln\left(\frac{w_{\mathrm{stamp}}}{R_{\mathrm{microtip}}}\right)-4.44\right]\frac{\gamma w_{\mathrm{stamp}}}{\bar{E}}}$$

(13) $$\begin{array}{l} h_{\max}=\sqrt{\left[5.14\ln\left(\frac{w_{\mathrm{stamp}}}{w_{\mathrm{microtip}}}-1.75\right)\right]\frac{\gamma w_{\mathrm{stamp}}}{\bar{E}}}\\ \times \exp\left\{0.371\sqrt{\frac{\gamma }{\bar{Ew_{\mathrm{stamp}}}}}{\left(\frac{w_{\mathrm{stamp}}}{w_{\mathrm{microtip}}}\right)}^{2}\times \left[1+\frac{2.08\;+\;\ln\left(\frac{P}{\sqrt{\gamma \bar{Ew_{\mathrm{stamp}}^{3}}}}\right)}{\sqrt{2.14\ln\left(\frac{w_{\mathrm{stamp}}}{w_{\mathrm{microtip}}}\right)-0.73}}\right]\right\} \end{array}$$

Figure 10E shows a graph of the seal size parameters. Where $w_{\mathrm{stamp}}$ is the width of the seal, $R_{\mathrm{microtip}}$ is the radius of the microtip, γ is the interface energy, $\bar{E}$ is the plane strain modulus, and P is the preload on the seal. The theoretical results and experimental results are in general agreement. Figure 10C,D shows experimental data graphs. Formulas (12) and (13) provide a theoretical basis for the design of the surface seal with a pyramid structure.

All of the above researchers used the PDMS seal. Due to the viscoelasticity of the PDMS seal, the microstructure gradually restores its original shape and releases the ink to the target substrate. Since the viscoelasticity of the PDMS seal is uncontrollable, the time during the release process is also uncontrollable. As a result, the transfer printing efficiency is too low, and the phenomenon of ink shedding often occurs. To solve this problem, Kim et al. [82] used SMP materials to prepare a pyramid microstructure seal and successfully solved the issue of seal release time. SMP materials can change shape with temperature changes and lock any shape and restore their original shape. This function can be used to reversibly change the SMP microstructure, and thus achieve the conversion of the seal adhesion strength. The specific process is shown in Figure 10F. Figure 10F(a) shows the rigid state of the seal. When the temperature is heated to T_g_ higher than the seal transition temperature to soften the seal, then the seal is contacted with the donor substrate, and a load is applied to collapse the microstructure on the surface of the seal. When the temperature of the seal is cooled below T_g_, the ink is extracted, the seal and ink are transferred to the top of the target substrate, and the seal is heated again until the original shape is restored.

### 2.4. Transfer Printing with an Applied Shear Load

**Figure 11:**
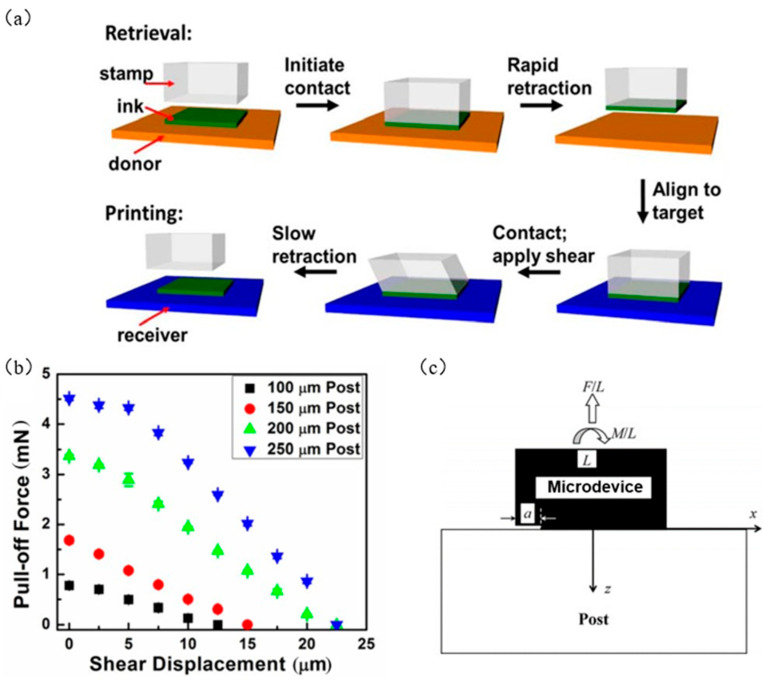
Transfer printing technology with an applied shear load. (**a**) Diagram of transfer with applied shear load (reproduced from [73]), (**b**) the relationship between shear displacement and adhesion (reproduced from [73]), and (**c**) transfer fracture mechanics model with shear load (reproduced from [84]).

In the process of printing ink to the target substrate, the relative adhesion strength between the ink and the seal interface must be less than the relative adhesion strength between the ink and the target substrate interface. In response to this requirement, Carlson et al. [73] found that applying a shear load on an elastic seal can produce a mixed load at the interface between the seal and the ink, thereby changing its fracture behavior [73]. When a certain shear displacement is applied, the adhesion between the seal and the ink can be reduced, and the ink can be printed on the target substrate. The specific process is shown in Figure 11a. Carlson et al. [73] also tested the seal adhesion under various shear loads. As shown in Figure 11b, when the stripping speed is 10 μm/s, for the four different seal sizes, as the shear displacement μ increases, the adhesive force gradually decreases.

Cheng et al. [84] established a fracture mechanics model for enhanced transfer with shear load, as shown in Figure 11c. In the process of separating the seal and the ink, it is assumed that a crack of the length is generated at the seal/microdevice interface, and the microdevice is under the combined action of the uniform pull force F/L and the uniform moment M/L, where $M=\tau hL^{2}=\mu \gamma hL^{2}$. The interface fracture form can be regarded as a mixture of type I and type II, and the simplified equations for the energy release rates of $K_{\mathrm{II}}$ and $K_{I}$ for type I and type II cracks are obtained:

(14) $$K_{I}=\sqrt{\pi L}\left(0.317\frac{F}{L^{2}}+1.285\frac{M}{L^{3}}\right)$$

(15) $$K_{\mathrm{II}}=\sqrt{\pi L}\left(0.103\frac{F}{L^{2}}+0.349\frac{M}{L^{3}}\right)$$

The energy release rate at the crack tip is obtained from the formula $G=\left(K_{I}^{2}+K_{\mathrm{II}}^{2}\right)/\left(2\bar{E}\right)$, where the plane strain modulus is $\bar{E}\approx 4E/3$, E is the elastic modulus, γ is the shear strain, h is the height of the seal, and L is the width of the seal:

(16) $$G=\pi EL\left[0.0417{\left(\frac{F}{EL^{2}}\right)}^{2}+0.0701{\left(\frac{\gamma h}{L}\right)}^{2}+0.108\frac{F}{EL^{2}}\frac{\gamma h}{L}\right]$$

When the crack energy release rate G reaches the fracture toughness $\Gamma_{0}$, the dimensionless relationship between the stripping force and the shear strain is obtained from the Griffith fracture criterion $G=\Gamma_{0}$ as:

(17) $$\frac{F}{EL^{2}}+\frac{13\gamma h}{10L}\approx 2\sqrt{\frac{6\Gamma_{0}}{\pi EL}}$$

**Figure 12:**
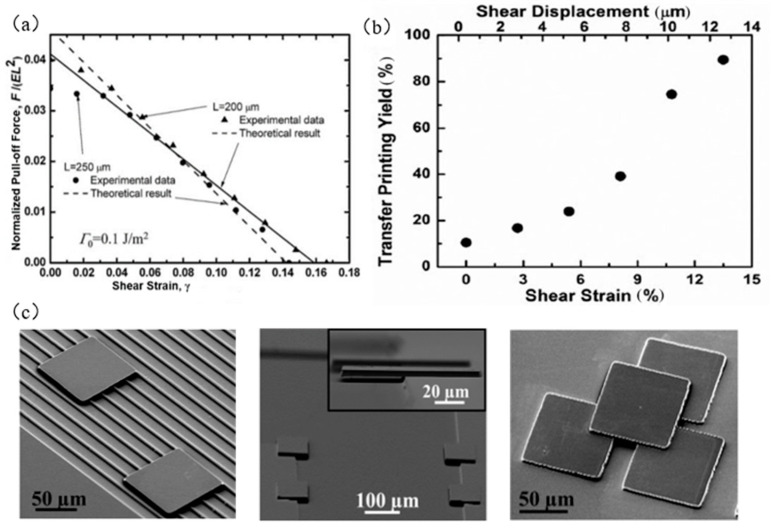
Principles and examples of transfer printing technology with an applied shear load. (**a**) The relationship between the dimensionless peel force and the shear strain *γ* (reproduced from [84]), (**b**) the relationship between shear strain *γ* and successful transfer area (reproduced from [73]), and (**c**) examples of small area target substrate printing, hanging structure, and multilayer structure transfer (reproduced from [73]).

Figure 12a shows the relationship between the dimensionless stripping force and the shear strain γ. It can be seen that the increase in shear strain can effectively reduce the stripping force of the interface, thereby reducing the adhesion force of the seal/device interface, to realize the transfer printing. Brendan et al. [83] improved the transfer printing technology that applied shear load and used this method to manufacture organic photovoltaic cells. This technology broke through the difficulty of printing relatively soft and large-area polymer films. It is particularly useful in organic electronics, where the morphology and interface structure of the film are critical to the performance of the device.

The transfer printing with applied shear load adopts physical means to apply shear load to the seal to realize the transfer (as shown in Figure 12b). The layer structure is shown in Figure 12c. However, because the seal needs a quantitative shear load, the transfer printing process is more complex, and to achieve a better transfer printing effect, a large shear load is needed, which may lead to the lateral sliding dislocation of the seal, and then affect the transfer accuracy, destroy the seal structure, and reduce the service life of the seal.

### 2.5. Tape-Assisted Transfer Printing

In the kinetic control transfer printing, due to the viscoelasticity of the seal, the stripping speed and the adhesive strength must be matched to achieve the transfer printing, but some instruments are still needed to complete it accurately. In addition, the adhesion strength is still high at every stripping speed. Based on the above reasons, researchers have developed tape-assisted transfer printing. The currently used tapes mainly include 3M tape [17,29,75], thermal release tape (TRT) [76,88], water-soluble tape (PVA) [75,77,89,90], and so on.

**Figure 13:**
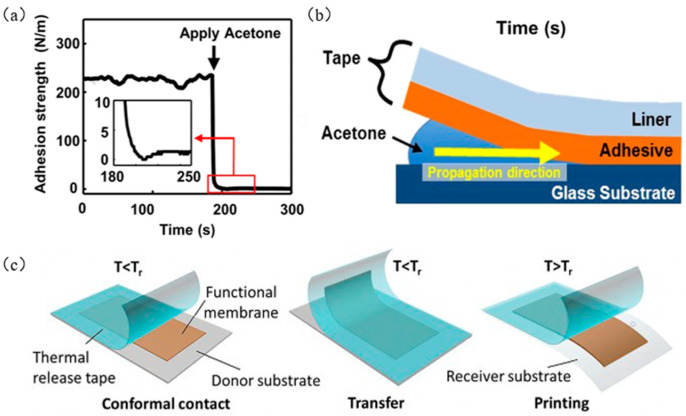
Tape-assisted transfer printing technology. (**a**) Change of 3M tape adhesion strength before and after adding acetone (reproduced from [75]), (**b**) schematic diagram of the competitive fracture of the tape interface (reproduced from [75]), and (**c**) heat release tape transfer printing diagram (reproduced from [76]).

**Figure 14:**
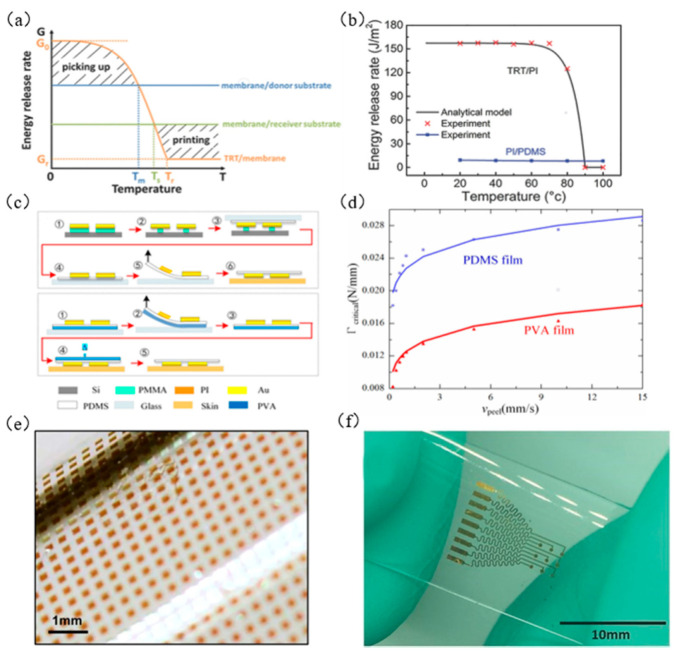
Principles and examples of tape-assisted transfer printing technology. (**a**) Graph of the relationship between temperature and interface energy release rate (reproduced from [76]), (**b**) graph of the relationship between the critical energy release rate and temperature at the interface of TPT/PI and PI/PDMS (reproduced from [76]), (**c**) diagram of adding PVA and adding PVA tape transfer printing (reproduced from [77]), (**d**) graph of the relationship between critical fracture energy and peeling speed (reproduced from [77]), (**e**) array of silicon particles transferred on the surface of a cylindrical tube (reproduced from [75]), and (**f**) nerve electrode array transferred to PDMS (reproduced from [76]).

Yu et al. [75] used methanol and propanol to chemically induce the commercially available 3M tapes, gradually reducing the high adhesion strength of the 3M tape, so that the ink was printed on the target substrate. Figure 13a shows the adhesion strength of 3M tape on glass before and after adding acetone at a stripping speed of 1 mm/min on the peel tester. As shown in Figure 13b, the tape consists of two layers, namely, tape and liner. Lin et al. [76] used TRT instead of the traditional seal (PDMS). By heating the TRT to a temperature higher than the transition temperature, the adhesion strength between the functional film and the TRT was weakened, resulting in the separation of the TRT and the functional film, thereby achieving transfer printing. The transfer printing process is shown in Figure 13c. Figure 14a shows the relationship between temperature (T) and interface energy release rate (G). Figure 14b shows the criticality of the TPT/PI and PI/PDMS interface graph of the relationship between energy release rate and temperature. Wang et al. [77] proposed an improved water-soluble tape transfer printing method based on polyvinyl alcohol, which can reduce the interface energy release rate by 60% compared with PDMS substrates, effectively helping to delaminate the glass surface. Figure 14c shows a flow chart of transfer printing without PVA and with PVA tape. Figure 14d presents a graph showing the relationship between critical fracture energy and stripping speed. Figure 14e shows a successful example of using 3M tape transfer printing, and Figure 14f shows a successful example of using TRT tape transfer printing.

Tape-assisted transfer printing has the technical characteristics of easy operation, large-area manufacturing, high yield, high fidelity, and the ability to transfer on curved surfaces, and the required tape manufacturing is simple and low in cost. However, this technology is often achieved through chemical treatment or physical heating, which may cause corrosion or damage to the transferred ink, leave residues on the ink, and contaminate the surface of the seal. Frequent replacement of the seal will reduce the performance of the equipment and ultimately affect the transfer effect.

### 2.6. Transfer Printing with Seal Inflatable

**Figure 15:**
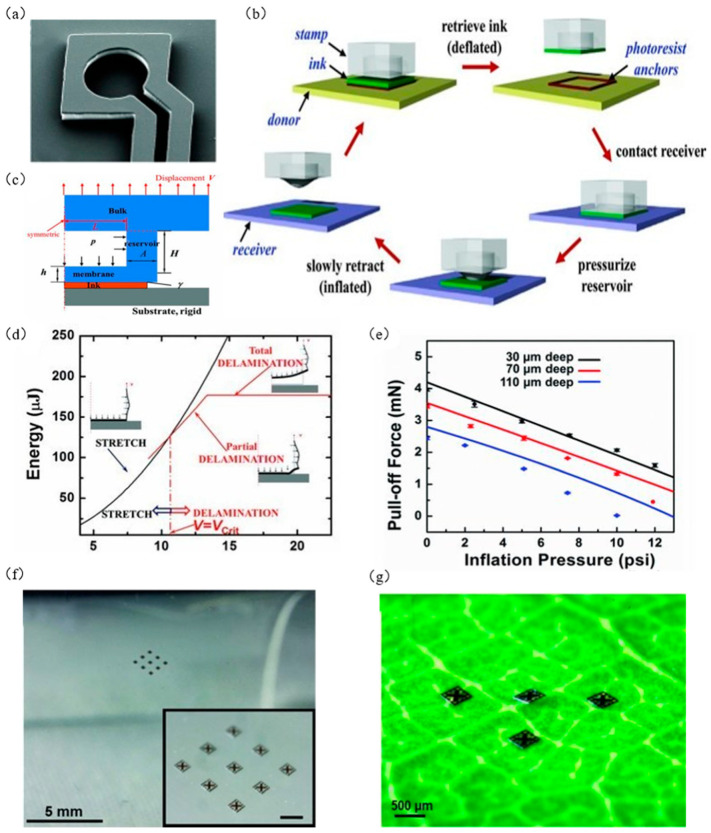
Transfer printing technology with inflatable seal. (**a**) Scanning electron microscope (SEM) of the inflatable seal (reproduced from [85]), (**b**) transfer printing diagram of seal inflation and expansion (reproduced from [85]), (**c**) mechanical model of inflatable seal (reproduced from [85]), (**d**) graph of the relationship between the displacement of the top of the seal and the energy (reproduced from [85]), (**e**) relationship between gas expansion pressure and tensile stress at different cavity depths (reproduced from [85]), (**f**) schematic diagram of 3 × 3 array silicon wafer transfer printing on PET target substrate (reproduced from [85]), and (**g**) schematic diagram of wafer transfer printing on blade (reproduced from [85]).

Carlson et al. [85], inspired by the adhesion and release process of aphid tentacles, designed an inflatable seal with an inflatable cavity structure using PDMS material and proposed a new method, which is a new method to control the free switching of the adhesion force during the transfer printing process by inflatable expansion/contraction. We call it transfer printing with an inflatable seal. Figure 15a shows a scanning electron microscope (SEM) image of an inflatable seal. Its main design features include a cavity for storing gas and a channel for circulating gas, as well as a PDMS film that seals the cavity and microchannels. Figure 15b shows a flow chart of transfer printing. The specific process for this method is: in the stage of ink extraction (the green sheet in Figure 15b), the seal is uninflated, the film is a flat surface, and the seal is in contact with the ink on a large area to achieve ink extraction. In the printing stage, the seal is inflated, and the film surface begins to expand, which reduces the contact area of the seal/ink and leads to a decrease in the adhesion in the seal/ink interface, making the ink transfer to the target substrate.

Carlson et al. [85] also established a mechanical model for inflatable seals (as shown in Figure 15c), assuming the cavity wall and the film as a beam structure. First, energy $U_{stretch}$ when the film falls off and energy $U_{delaminate}$ when the film does not fall off were calculated, and displacement V was applied on the top of the seal to make the displacement $V_{\mathrm{crit}}$ when the film falls off. When $V<V_{\mathrm{crit}}$,$U_{stretch}<U_{delaminate}$, the film will not separate from the seal. When $V>V_{\mathrm{crit}}$, $U_{stretch}>U_{delaminate}$, the film will separate from the seal. When $V=V_{\mathrm{crit}}$ (as shown in Figure 15d), the maximum tensile stress is

(18) $$P_{\mathrm{pull}-\mathrm{off}}=\frac{4E_{R}AV_{\mathrm{crit}}\left(L+A\right)}{H}-p\pi L^{2}$$

where *E_R_* is the elastic modulus of the seal, *A* is the width of the cavity wall, *L* is the general width of the film, *H* is the depth of the cavity, and *p* is the evenly distributed gas expansion pressure inside the cavity. Figure 15e shows the relationship diagram between gas expansion pressure and tensile stress at different cavity depths measured by experimental data. It can be seen that the experimental results are consistent with Formula (18), so the seal can be transferred by controlling the air pressure in the cavity.

Figure 15f shows the transfer printing of 3 × 3 array silicon wafers on the PET target substrate, and Figure 15g shows the transfer of silicon wafers on the blade in a rough nonplanar state. These examples show that the transfer printing of the inflatable seal enables free switching of adhesion force and programmable deterministic assembly and does not cause damage to the ink and the seal. However, due to the dimensional accuracy requirements of the cavity and the microchannel, the manufacturing process is complicated and the cost is high.

### 2.7. Magnetic Control Transfer Printing

**Figure 16:**
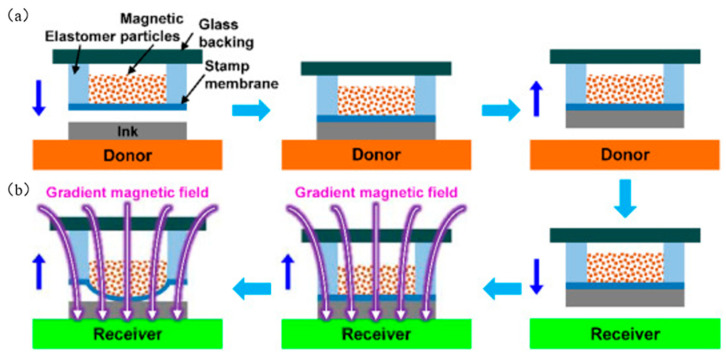
Diagram of magnetic controlled transfer printing technology (reproduced from [56]), (**a**) Represents the process of extracting ink from the source substrate by the magnetic seal, and (**b**) Represents the process of printing the magnetic seal on the target substrate.

**Figure 17:**
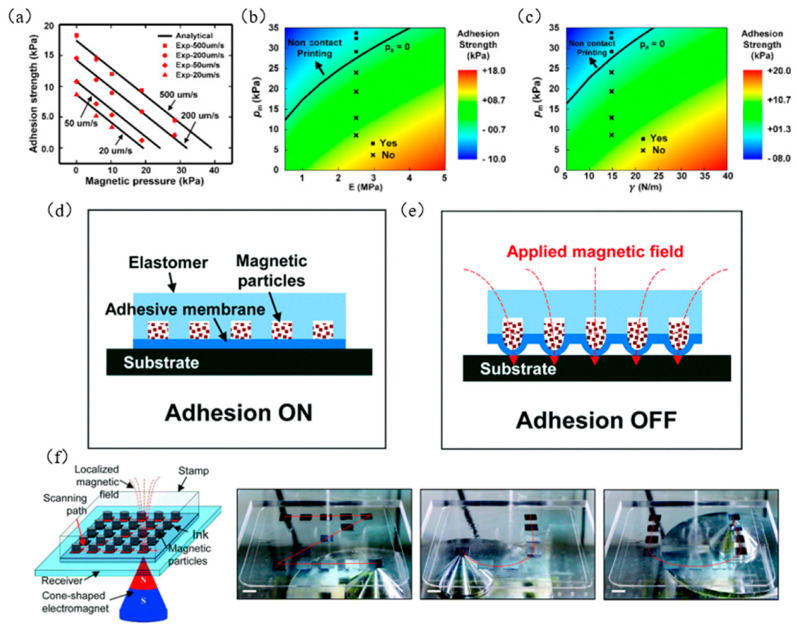
Principles and examples of magnetic control transfer printing technology. (**a**) Graph of the relationship between magnetic pressure and adhesion strength at different retraction speeds (reproduced from [56]), (**b**) diagram of the relationship of seal elastic modulus (E) and magnetic pressure (p_m_) (reproduced from [56]), (**c**) the relationship between adhesion strength (γ) and magnetic pressure (p_m_) (reproduced from [56]), (**d**) schematic diagram of strong adhesion state (reproduced from [72]), (**e**) schematic diagram of weak adhesion state (reproduced from [72]), and (**f**) examples of successful transfer printing using magnetic field control (reproduced from [72]).

Song et al. [72] developed a magnetic control transfer printing method by exploiting the advantage of the fast response of magnetic particles in a magnetic field, as shown in Figure 16. This method provides rapid adjustment of adhesion, and the adhesion is reversible, which provides a new method for deterministic assembly. Li et al. [56] further studied the influence of magnetic pressure (p_m_) and seal elastic modulus on adhesion strength. Figure 17a shows the relationship between magnetic pressure and adhesion strength at different retraction speeds. The adhesion strength increases with the increase in the retraction speed; with the increase in magnetic pressure, the adhesion strength will gradually decrease. When the magnetic pressure is large to a certain extent, the adhesion strength becomes zero, thus achieving noncontact transfer printing. Figure 17b,c shows the influence of elastic modulus (E), adhesion strength (γ), and magnetic pressure (p_m_) of the seal when the retraction speed is 100 μm/s. The adhesion strength changes from positive to negative when the magnetic pressure is greater than the critical value, which means that the force exerted by the seal on the ink changes from an adsorption force to a release force. The black solid line (P_a_ = 0) in Figure 17b,c represents the critical state of noncontact transfer printing, and the design parameters of magnetic pressure and elastic modulus in noncontact transfer printing are determined. Song et al. [72] were inspired by aphid tentacles to design a magnetically driven elastomeric seal, which has an array structure that can switch between strong and weak adhesion state in a fast and reproducible manner, as shown in Figure 17d,e. It also realized the transfer printing of silicon wafers to ceramic substrates in air and vacuum, which demonstrates the potential of magnetic control transfer printing in the semiconductor and display industries. Figure 17f shows an example of successful transfer printing using magnetic field control, which illustrates that magnetic field control transfer achieves selective printing.

Magnetic control transfer printing technology can realize selective printing, which has the characteristics of rapid adjustment of adhesion force and deterministic assembly. However, due to the thin film thickness of the sealing seal, when the magnetic particles are affected by the magnetic field for a long time, the film is easy to be damaged, even cracked.

## 3. Conclusions and Outlook

This review discusses seven main transfer methods in the field of transfer printing. For each method, a detailed overview of its working principle, the mechanical model of the seal, and the influence of relevant parameters on the transfer printing is shown, and some examples of the successful transfer by each method are shown. Finally, the technical characteristics and defects of each transfer printing method are summarized at the end of each section.

Finally, based on the development of the above transfer printing methods, it is possible to conclude the outlook for future work in the field of transfer printing as a whole. First, the mechanism of each microtransfer printing method is studied. Currently, fracture mechanics is usually used to study the mechanism of microtransfer printing, but there is a need for more accurate modeling methods because of various research factors. Second, for the design of a seal with microstructures, this kind of seal is usually used together with other transfer printing methods, but the influence of the size design of the seal surface microstructure on the transfer is often neglected. At present, the seal materials are PDMS and SMP, so basically, there is a need for more superior performance materials as transfer printing seals. Finally, and the most important point, the current transfer printing technology is mostly limited to scientific experimental research and cannot be applied to industrial production, which deviates from the original purpose of micro-transfer printing technology. In the future, we can carry out microtransfer printing technology for industrial application research and exploration.

## Figures and Tables

**Table 1:** Comparison of seal adhesion force with different characteristics.

Seal Category	Seal Material Composition	Elastic Modulus	Glass Transition Temperature	Seal Surface	Maximum Adhesion	Minimum Adhesion	Adhesion Control Mechanism	Corresponding Transfer Method	References
PDMS	By weight ratio of 10: A prepolymer of 1 is mixed with a crosslinker	1.32–2.97 MPa	−125 °C	Flat seal	120	74	Control seal peeling speed	Kinetic control transfer printing	[70]
				Flat seal	1530	128			[71]
			Below 100 °C	Microstructural seal of tiny structure	312.5	≈0.3	Thermal mismatch occurs between interfaces during laser heating	Laser control seal temperature transfer printing	[4,39]
			−125 °C	Flat seal	22	Almost 0	Magnetic force is used to control the swelling and collapse of the film to achieve the switch between the seal and ink contact area	Magnetic control transfer printing	[72]
	The weight mixing ratio of the prepolymer to the crosslinker is 5:1	1.8–2.1 MPa		Pyramidal microstructure	80	0.08	By changing the seal and ink contact area	Microstructure seal to assist in transfer printing	[61]
				Oblique column microstructure	100	1			[62]
				Flat seal	85	8.5	Apply a shear load on the seal to adjust the adhesion	Transfer printing with applied shear load	[73]
SMP	By weight ratio of 45: 23 epoxy monomer E44 and Jeffamine D230		40–60 °C	Flat seal	3200	534	Thermal mismatch occurs between interfaces during laser heating	Laser control seal temperature transfer printing	[57,68]
				Pyramidal microstructure	2800	2.8			
CBSMP	The mole ratio is 1:1:1 EPON 826 (heated-out impurity), Jeffamine D230, and NGDE, which were mixed, and XC72R powdered carbon black was used as additive						Thermal mismatch occurs between interfaces during laser heating	Laser control seal temperature transfer printing	[74]
3M 3850 tape				Flat seal	880	Almost 0	By adding acetone to control the switch of adhesion	Tape-assisted transfer printing	[75]
Thermal release tape (TRT)			100 °C	Flat seal			By heating the TRT tape, the adhesive force is irreversibly reduced	Tape-assisted transfer printing	[76]
Water-soluble tape (PVA)	Polyvinyl alcohol	1 GPa		Flat seal			Use PVA water-soluble tape as a sacrificial layer to achieve the effect of reducing the interfacial adhesion energy	Tape-assisted transfer printing	[77]
